# Hyperestrogenic imprinting and developmental origins of endometriosis and adenomyosis

**DOI:** 10.1093/hropen/hoag075

**Published:** 2026-08-10

**Authors:** Sun-Wei Guo, Paola Viganò

**Affiliations:** Research Institute, Shanghai OB/GYN Hospital, Fudan University, Shanghai, China; Shanghai Key Laboratory of Reproduction and Development, Fudan University, Shanghai, China; Shanghai Key Laboratory of Female Reproductive Endocrine-Related Diseases, Fudan University, Shanghai, China; Infertility Unit, Fondazione IRCCS Ca’ Granda Ospedale Maggiore Policlinico, Milano, Italy

**Keywords:** adenomyosis, developmental origins, endocrine-disruptive chemicals, endometriosis, hyperestrogenic imprinting, *in utero*, neonatal, pathogenesis, xenobiotic estrogens

## Abstract

The pathogenesis of endometriosis and adenomyosis remains incompletely understood, and a notable gap persists between the reported heritability and the disease variation explained by identified susceptibility loci. In this manuscript, we showcase key epidemiological and clinical observations—including associations with early menarche, shorter anogenital distance, specific childhood growth trajectories, hypospadias in males, and developmental exposure to endocrine-disrupting chemicals—that collectively point to the influence of early-life developmental programming. Recent evidence revealing a hyperestrogenic/hypoandrogenic hormonal imbalance in the umbilical cord blood of pregnant women with endometriosis provides a critical link between these seemingly disparate findings. We propose a novel hypothesis of hyperestrogenic imprinting: Female offspring of women with endometriosis and/or adenomyosis are exposed *in utero* to a distinct hormonal environment, which induces lasting epigenetic changes in the developing reproductive tract, thereby imprinting a lifelong susceptibility to these two diseases. This mechanism offers a cohesive explanation for the familial aggregation of endometriosis and possibly adenomyosis, addresses the issue of ‘missing heritability’, and integrates various risk factors rooted in early development. A limitation of this opinion piece is that its hypothesis is primarily informed by animal studies and conceptual synthesis, and would benefit from further validation through original human data. As a falsifiable hypothesis, it presents a clear pathway for future validation and, if proven, opens avenues for novel preventive strategies.

## Introduction

Despite decades of intensive research, the pathogenesis of endometriosis and its associated disease, adenomyosis, remains only partially understood. Although Sampson’s theory of retrograde menstruation (Sampson, 1927; Viganò *et al.*, 2024) remains the most widely accepted explanation for endometriosis, it alone cannot account for the full complexity of the disease, particularly its age-specific prevalence and the plethora of endocrine and molecular factors involved. This shortcoming underscores the need for continued investigation into intersecting mechanisms (Endometriosis Initiative, 2024). For adenomyosis, the hypothesis of endometrial–myometrial interface disruption (EMID) (Guo, 2020) is supported by ample epidemiological data showing that a history of uterine procedures is a strong risk factor (Levgur *et al.*, 2000; Curtis *et al.*, 2002; Chapron *et al.*, 2020). Additional support comes from animal experiments in which EMID procedures, designed to mimic uterine procedures in humans, successfully induce adenomyosis (Hao *et al.*, 2020; Elsherbini *et al.*, 2022; Guo *et al.*, 2025). However, not all adenomyosis cases result from iatrogenic uterine procedures (Kishi *et al.*, 2012; Guo, 2020), indicating that other pathogenic pathways must exist. In this opinion article, we propose a novel hypothesis for the pathogenesis of endometriosis and adenomyosis.

## Heritability and missing heritability of endometriosis

Endometriosis has long been recognized to show familial aggregation, with increased risk among first-degree relatives and sisters (Bischoff and Simpson, 2000; Matalliotakis *et al.*, 2008). A frequently cited estimate of the contribution of genetic factors to the pathogenesis of endometriosis is a heritability of 47–51% (Treloar *et al.*, 1999; Saha *et al.*, 2015), which to many implies that nearly half of the causes for endometriosis are determined by heritable genetic susceptibility.

However, a recent study involving tens of thousands of subjects reports that all 42 loci identified to date explain only ∼2% of disease variance (Rahmioglu *et al.*, 2023). Using the 47–51% heritability estimates cited above, this finding means that more than 95.7–96.1% of the estimated heritability remains unaccounted for, or ‘missing’. While the presence of gene–gene interaction (GGI), gene–environment interaction (GEI), and structural variants could be responsible for this missing heritability, it should be noted that both the narrow-sense and broad-sense heritability is defined under the assumptions of no GGI and no GEI (Guo, 1999). In other words, in the presence of GGI and/or GEI, the heritability cannot be properly defined since it would lead to either mathematical intractability when there is GGI or difficulty in attributing nature versus nurture in the presence of GEI due to inextricable gene and environment effects. Although modern approaches such as genome-wide complex trait analysis have advanced our ability to model certain interaction components (Yang *et al.*, 2010; de Los Campos *et al.*, 2015), these methods still face substantial challenges in fully partitioning GGI and GEI within linear frameworks, particular when not all major genetic or environmental factors have been identified. Consequently, traditional heritability estimates may be limited in their capacity to capture the full complexity of non-additive genetic and environmental interplay. This limitation underscores the value of shifting focus from static sequence variants to dynamic regulatory alterations, such as epigenomic modifications and tissue-specific enhancer activity, which may better reflect the developmental programming underlying endometriosis and adenomyosis susceptibility. Indeed, a substantial portion of identified endometriosis-predisposing genetic variants are located in the non-coding regions (Sapkota *et al.*, 2017), and many of them are found to possess tissue-specific regulatory capabilities (Garibaldi-Rios *et al.*, 2025). A non-coding variant that affects gene regulation is very likely to be influenced by environmental cues, often more so than a variant that alters the protein-coding sequence itself. These data seem to implicate the presence of GEI in endometriosis, consistent with epidemiologic data linking endometriosis and, say, red meat consumption (Yamamoto *et al.*, 2018).

Regardless, these 40+ loci likely represent the low-hanging fruits, i.e. genetic variants conferring larger effect sizes. Therefore, the identification of any additional loci, if they exist, is expected to be progressively more challenging, as each additional variant would likely confer only an infinitesimal risk and thus a miniscule increment in disease risk. In light of these findings, one may wonder: If the identified genetic variants do not fully account for familial aggregation, why does familial aggregation exist in the first place?

Of course, even in the complete absence of any genetic factor, shared environmental exposure (such as diet, exposure to endocrine-disrupting chemicals (EDCs), socioeconomic factors, shared microbiome, or shared lifestyle factors) alone could account for the observed familial aggregation of endometriosis (Guo, 2000). However, the reported vertical transmission pattern from large pedigree analysis (Stefansson *et al.*, 2002) seems to suggest something more than just familial aggregation of risk factors. What are they, then?

## Hyperestrogenic imprinting

The tendency of familial aggregation in endometriosis, and possibly in adenomyosis as well, could be explained, at least in part, by a mechanism we term *hyperestrogenic imprinting* (HEI). We define HEI as the process by which developmental, particularly *in utero*, exposure to hyperestrogenism—whether maternal (due to endometriosis and/or adenomyosis) or environmental (e.g. xenobiotic estrogens)—induces persistent and lasting epigenetic and estrogen signaling alterations in the uterus and the pelvic cavity, thereby increasing the risk of developing endometriosis and/or adenomyosis. Similar to genetic imprinting, which is essentially an epigenetic mechanism, prenatal and neonatal exposure to increased estrogens may trigger a cascade of events leading to alterations in epigenome and estrogen signaling in the reproductive tract of female offspring. These changes, in turn, may increase the risk of developing the diseases and potentially contribute to the apparent familial aggregation.

Growing evidence indicates that fetal and neonatal development is critical in shaping adult life in both animals and humans. One striking example is the early consumption of royal jelly in honeybees: all larvae consume royal jelly for the first 3 days of their lives, but worker bees switch to worker jelly thereafter, while the queen bee larvae continue consuming royal jelly as her main food source into adulthood. This dietary difference, coupled with the provision of a queen cell (Fang *et al.*, 2026), results in two morphologically and reproductively divergent castes: infertile workers versus the fertile queen, driven by royal jelly-induced epigenetic changes despite identical genomes (Alhosin, 2023). Thus, the difference in early-life diet and living condition completely change reproductive destiny.

Furthermore, the vindication of Barker’s hypothesis and related research illustrates how maternal environment can tune the offspring epitranscriptome, with profound and long-lasting health consequences. Paradigmatic examples include maternal overnutrition, which leads to offspring with elevated incidence of obesity, increased food intake, impaired glucose tolerance, and elevated incidence of cardiovascular disease (Guillaumin and Peleg-Raibstein, 2023). Increased maternal physical activity enhances metabolic health (Sheldon *et al.*, 2016; Quiclet *et al.*, 2017; Stanford *et al.*, 2017), cognitive function (Yang *et al.*, 2021), and physical performance (Beleza *et al.*, 2021) in offspring. Paternal exercise confers endurance capacity to offspring through epigenetic mechanisms (Yin *et al.*, 2025). These data uncannily exhibit some form of imprinting, involving epigenetics. Further, maternal effects and epitranscriptomic regulation have been proposed to intersect into a unified system of non-genetic developmental programming (Ahi, 2026).

In the following sections, we present supporting data and build a case for this HEI hypothesis for the pathogenesis of endometriosis and/or adenomyosis. We also outline ways to test this hypothesis.

## Clues from early-life associations

### Early age at menarche and endometriosis/adenomyosis

A recent meta-analysis of 16 papers published between 2000 and 2020 demonstrated that early menarche (< 12 years) is associated with a significant increased pooled risk of endometriosis (odds ratio (OR) = 1.34, 95% CI = 1.16–1.54) (Lu *et al.*, 2023). According to the California Teachers Study, early menarche (≤10 years of age) is statistically significantly associated also with adenomyosis (Templeman *et al.*, 2008).

Earlier age at menarche influences both the frequency of menstruation and duration of menstrual exposure, which aligns with Sampson’s theory. Therefore, determinants of age at menarche—whether modifiable or not—may indirectly influence disease risk by creating a prolonged period of exposure to ‘incessant menstruation’, a recognized strong risk factor for endometriosis (Vercellini *et al.*, 2011).

Population differences in age at menarche are influenced by genetics, lifestyle, and environmental exposures (Dong *et al.*, 2023), as well as early-life factors such as birthweight, gestational age, and developmental exposure to EDCs (Watkins *et al.*, 2017). Thus, a key question emerges: what are the specific determinants of early age at menarche in women with endometriosis/adenomyosis?

### Shorter anogenital distance and endometriosis/adenomyosis

Anogenital distance (AGD), defined as the distance between the anus and the genital tubercle, is a sexually dimorphic trait that is typically longer in male mammals but shorter in females (Mendiola *et al.*, 2016; Garcia-Penarrubia *et al.*, 2020). It reflects androgen exposure levels *in utero* during the masculinization programming window (gestational weeks 8–14) and is considered a lifelong marker of the prenatal hormonal environment (Welsh *et al.*, 2008; Schwartz *et al.*, 2019) as well as of prenatal exposure to endocrine disruptors (Bornehag *et al.*, 2015; Swan *et al.*, 2015).

Shorter AGD has been reported to be associated with a higher risk of endometriosis, especially deep endometriosis. A meta-analysis including 1033 subjects found a significant difference between women with endometriosis and controls for AGD measured from the anus to the posterior fourchette (standard mean difference = −0.87, *P* = 0.0096) (Crespi, 2024). Shorter AGD is also associated with a higher risk of adenomyosis but not uterine fibroids (Liu *et al.*, 2023).

The link between shorter AGD and endometriosis/adenomyosis raises critical questions: Why is a shorter AGD associated with a higher risk of developing these diseases? Could *in utero*—or perhaps perinatal—exposure to higher estrogens and/or lower androgen levels increase this risk?

### Growth trajectory and endometriosis

The inverse relationship between BMI and endometriosis has long puzzled researchers, as it seems inconsistent with the expectation that an estrogen-dependent disease like endometriosis would be promoted by increased adiposity and thus higher estrogen levels. However, body size during childhood and adolescence, around the time of menarche, appears to be a more relevant exposure than body size at the time of diagnosis or investigation. Using data collected from the Nurses’ Health Study II, including 1817 cases with laparoscopically confirmed endometriosis, Vitonis and colleagues demonstrated that body size at ages 5, 10, and 20 were all inversely associated with the disease. Specifically, the relative risks (RRs) for endometriosis in females who had the smallest figures, compared with the middle category, were 1.23 (95% CI = 1.08–1.40) for size at age 5 and 1.18 (95% CI = 1.02–1.36) during childhood (ages 5–10 years) (Vitonis *et al.*, 2010).

Importantly, sex steroid hormone levels strongly contribute to offspring growth and adiposity both *in utero* and postnatally. Estrogen and testosterone levels in pregnancy can have a strong impact not only on birthweight and neonatal adiposity but also on infant growth trajectories (Meng *et al.*, 2025). This connection prompts another question: How might childhood growth patterns, occurring before the onset of menstruation, contribute to the later development of endometriosis?

### Congenital anomalies in offspring born from women with endometriosis

A recent study including 1.46 million births found that congenital anomalies were more common in infants of mothers with endometriosis than in controls, with one of the strongest associations observed for hypospadias (adjusted RR = 1.47, 95% CI = 1.04–2.09) (Milne *et al.*, 2026). Only about 11% of this increased risk was explained by assisted reproductive technologies, while other fertility treatments did not meaningfully mediate the association (Milne *et al.*, 2026).

Hypospadias, a misplacement of the urethra meatus, results from a failure of urethral closure during development. Both in mice and humans, any disruption to hormonal signaling in early development can cause hypospadias. More specifically, estrogenic compounds and those that antagonize the androgen receptor or inhibit fetal testis steroidogenesis, reduce testosterone production and/or its action to cause male reproductive tract malformations, including hypospadias (Mattiske and Pask, 2021).

Here the question is: Could *in utero* or perhaps perinatal exposure to higher estrogen and/or lower androgen levels increase the risk of hypospadias in the offspring of women with endometriosis?

### Developmental exposure to xenobiotic estrogens and the risk of endometriosis/adenomyosis

Abundant research has demonstrated that the fetus and neonate are in a critical developmental period, and their reproductive tracks are exquisitely sensitive to EDCs, especially xenobiotic estrogen (Yamashita, 2006). Extensive animal studies provide compelling and consistent evidence of a causal relationship between prenatal and neonatal exposure to estrogens and xenobiotic estrogens and adenomyosis development.

Prenatal exposure to:

- diethylstilbestrol (DES) results in lasting effect on the reproductive tract, including adenomyosis (Huseby and Thurlow, 1982; Newbold *et al.*, 2007a; Yin *et al.*, 2012; Jefferson *et al.*, 2013);
- 2,3,7,8-tetrachlorodibenzo-p-dioxin causes adenomyosis in mouse, and this effect appears transgenerational (Bruner-Tran *et al.*, 2016);
- bisphenol A (BPA) enhanced the sensitivity to provoked pain in rats, suggestive of changes in the activity of neural pathways and/or centers involved in nociception and pain perception (Aloisi *et al.*, 2002). Because pain is a potent inducer of stress, the elevated pain sensitivity may activate the hypothalamic–pituitary–adrenal axis and promote lesional development through adrenergic receptor β2 (Long *et al.*, 2016; Guo *et al.*, 2017);

Neonatal exposure to:

- DES appears to result in permanent DNA methylation and gene-specific changes in histone modification enzymes in the uterus, likely causing widespread changes in gene expression in adulthood (Li *et al.*, 2003; Newbold *et al.*, 2007a; Tang *et al.*, 2008; Jefferson *et al.*, 2013);
- BPA (Newbold *et al.*, 2007b), tamoxifen (Parrott *et al.*, 2001), 17β-estradiol (E_2_) (Singh and Bhartiya, 2023) or even an ERβ agonist (Cao *et al*., 2022) causes adenomyosis in adult mice, and also causes preferential epigenetic programing of estrogen response (Jorgensen *et al.*, 2016). In particular, neonatal tamoxifen exposure in female ICR/CD-1 mice not only induces adenomyosis but also sears a seemingly lasting epigenetic imprint on the reproductive tract (Chen *et al.*, 2026).

While rodent data on developmental exposure to xenobiotic estrogens and endometriosis risk are lacking since rodents do not develop endometriosis spontaneously, epidemiological studies indicate that fetal exposure to DES in humans is associated with an increased risk of endometriosis. One prospective study reported an 80% higher incidence (RR = 1.8, 95% CI =1.2–2.8) (Missmer *et al.*, 2004), while a later case-control study found an increased risk of endometriosis by 100% but the confidence interval includes the null (OR = 2.0, 95% CI =0.8–4.9) (Upson *et al.*, 2015).

In 2021, Gaspari *et al.* described a multigenerational Caucasian family with endometriosis across two consecutive generations. Although the mother had no history of endometriosis, she received high-dose DES after each delivery to suppress lactation. Her first daughter, unexposed to DES, remained unaffected, whereas her six subsequently exposed daughters all developed laparoscopically confirmed endometriosis. In the third generation, all seven daughters of affected women had imaging-confirmed endometriosis, while none of the daughters of the unexposed woman were affected (Gaspari *et al.*, 2021). While this unusual clustering might still be dismissed as anecdotal due to its highly selective nature, the findings are nonetheless consistent with earlier epidemiological findings (Missmer *et al.*, 2004; Upson *et al.*, 2015).

The global epigenetic changes resulting from developmental exposure to EDCs, especially xenobiotic estrogens, could account for long-lasting, persistent, and likely profound transcriptional aberrations in the reproductive tract due to downstream gene alterations (Bredfeldt *et al.*, 2010; Jefferson *et al.*, 2013). This leads to a final synthesizing question: Which underlying mechanistic processes are common to the various strategies used to induce adenomyosis in rodent models and between these and the epidemiological findings supporting an association of endometriosis with *in utero* exposure to synthetic estrogens?

## Finding the missing link

**Figure 1.**
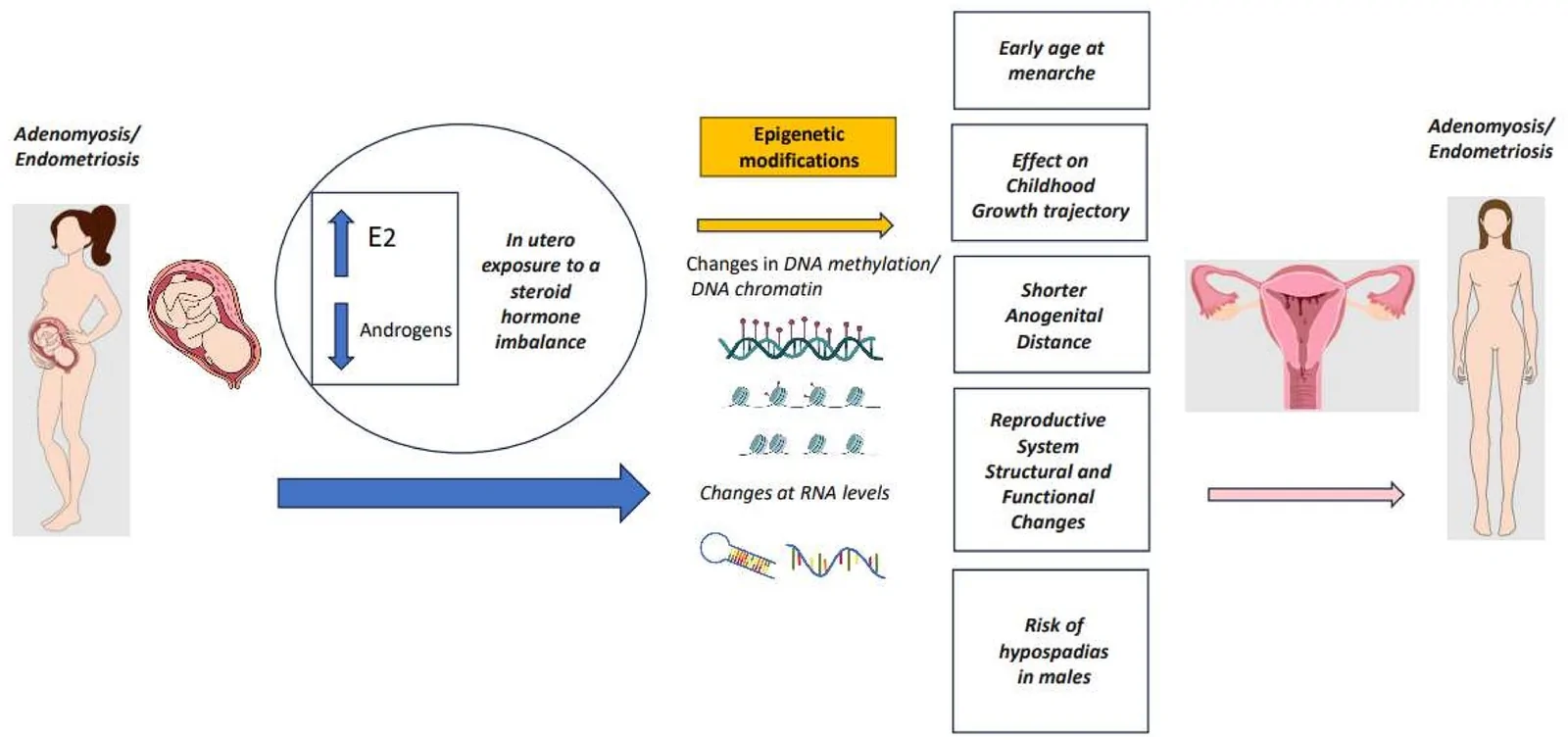
**Schematic diagram showing the main features of the hyperestrogenic imprinting hypothesis.**  *In utero* exposure to an hyperestrogenic/hypoandrogenic hormonal milieu induces lasting epigenetic changes in the developing reproductive tract. This imprint leads to several phenotypic manifestations including early age at menarche, a shorter anogenital distance, a particular childhood trajectory and an increased risk of hypospadias in male neonates, and, as such, confers a lifelong susceptibility to endometriosis and/or adenomyosis upon exposure to permissive secondary factors such as retrograde menstruation. E2, 17β-estradiol.

The hormonal imbalance occurring during prenatal period and possibly neonatal period as well represents a unifying explanation for these observations and possible answers to the issues raised. The recent work by Salmeri and colleagues provides a critical piece of evidence. In a case-control study of women delivering singleton females, they reported that female newborns of women with endometriosis are exposed *in utero* to an imbalance in estrogen/androgen levels compared to controls without the disease (Salmeri *et al.*, 2025). Cases were women with endometriosis diagnosed by imaging or surgery before pregnancy; controls were women without endometriosis. Umbilical cord blood samples were collected at delivery, and levels of steroid hormones were measured using liquid chromatography–tandem mass spectrometry. Analyses were performed before and after propensity score matching (PSM) at a 1:3 ratio to control for maternal age, gestational age, and delivery mode. Women with endometriosis had higher E_2_ levels than controls, both before matching (6.8 [4.3–9.1] mcg/l vs 3.6 [1.4–8.6] mcg/l, *P* = 0.03) and after matching (2.4 [1.1–6.6] mcg/l, *P* = 0.002) (Salmeri *et al.*, 2025). After PSM, the E_2_-to-testosterone and E_2_-to-androstenedionewere 4.38 (95% CI = 2.28–6.49) and 3.28 (95% CI= 1.29–5.28) times higher in cord blood of women with endometriosis. These findings directly link existing epidemiological clues and animal exposure studies to a measurable hormonal perturbation in women with endometriosis. This altered milieu would have potential long-term consequences for the offsprings. Figure 1 illustrates the main feature of this hypothesis.

It should be noted that since the blood samples in this study were taken at the delivery, it is unclear whether the high estrogen-to-androgen ratio in the cord blood is present during the entire gestational period or just a one-time event occurring only at the time of delivery. In addition, this observation is based on a single case-control study with inherent design limitations, and, as such, requires independent validation. Therefore, further longitudinal human studies or cohort studies using biobank samples measuring maternal serum/amniotic fluid hormones during early-to-mid gestation are required to fully validate whether this hyperestrogenic state is sustained throughout pregnancy.

## Limitations and caveats

To a large extent, our hypothesis is built upon extensive animal experimentation, which should be acknowledged as a limitation with regard to its translational validity. On the other hand, while rodents and humans differ greatly in physiology, a lot of mechanisms are well-conserved among mammals. For example, AGD, which mirrors the *in utero* exposure levels of androgen, was first discovered in rodents but later found to be also true in humans.

Another paradigmatic example comes from variations in maternal care in rats. A landmark study showed that differences in early maternal caregiving behaviors during the first week of life could alter DNA methylation of the *Nr3c1* (coding for glucocorticoid receptor, GR) promoter in the offspring’s hippocampus (Weaver *et al.*, 2004). Higher levels of maternal care reduced *Nr3c1* methylation, leading to increased GR expression, improved cortisol feedback, and greater stress resilience (Weaver *et al.*, 2004; Meaney and Szyf, 2005). Similar effects have been observed in humans, where the homologous *NR3C1* promoter is influenced by early-life factors such as maternal depression, caregiving quality, breastfeeding, and prenatal stress. Lower *NR3C1* methylation is associated with better stress regulation, whereas higher methylation is linked to greater cortisol reactivity and increased risk of stress-related disorders, including depression (Veltri *et al.*, 2025).

Another reason for caution refers to the inherent limitations of epidemiological association studies, which are susceptible to confounding, selection bias, and information biases (including recall bias), thereby limiting causal inference.

## The need for further investigation

Very importantly, this HEI hypothesis is falsifiable. By carefully designing animal experiments and human studies, this hypothesis can be rigorously tested in different ways. For example, taking advantage of the fact that rodents can spontaneously develop adenomyosis, one could evaluate whether *in utero* exposure to exogenous estrogens would result in shortened AGD and increased risk of developing adenomyosis. Conversely, one could manipulate the *in utero* estrogen-to-androgen ratio to create a hyperandrogenic environment (Wang *et al.*, 2026) and see whether the risk of adenomyosis can be reduced. In addition, single-cell RNA sequencing could be employed to identify distinct cell populations with distinct expression profiles within the uterus and/or pelvic cavity, followed by further elucidation of their roles and functions in inducing adenomyosis. Alternatively, one could build on the previous reports on the permanent epigenetic alterations in genes/proteins involved in epigenetic regulation and estrogen signaling in the reproductive tract of mice developmentally exposed to xenobiotic estrogens such as DES (Jefferson *et al.*, 2013). These same genes/proteins could be investigated in mice developmentally exposed to hyperestrogenism to further elucidate their roles, if any, in the induction of adenomyosis. In parallel, longitudinal studies following female offspring born to mothers with and without endometriosis/adenomyosis could help test this hypothesis by comparing the incidence of these conditions. Additional supporting evidence could be obtained by assessing AGDs in female offspring of affected versus unaffected mothers. These epidemiological studies are highly feasible in light of publications of some cohort studies on the incidence of endometriosis and/or adenomyosis based on the national registry (Magnus *et al.*, 2026). Incidentally, this study reports higher risk of endometriosis and/or adenomyosis in female offspring born to mothers undergone assisted reproductive technologies, in which estrogen was often administrated during the first few weeks of gestation for frozen embryos. Thus, their finding of higher risk of endometriosis/adenomyosis is potentially compatible with the HEI hypothesis.

## Conclusion

We propose that HEI may account for a significant component of the pathogenesis of endometriosis and adenomyosis. Specifically, women with endometriosis and/or adenomyosis have an imbalance in estrogen/androgen levels in their cord blood during pregnancy. Their female offspring are therefore exposed to a distinct, hyperestrogenic/hypoandrogenic environment *in utero*, which induces lasting epigenetic changes in the developing reproductive tract. This imprint confers a lifelong susceptibility, making the offspring more susceptible to developing endometriosis and/or adenomyosis upon exposure to permissive secondary factors such as retrograde menstruation. Conceivably falsifiable, this hypothesis provides a cohesive explanation for the familial aggregation of endometriosis and possibly adenomyosis, as well as for the ‘missing heritability’. If validated, it would raise the possibility of preventive interventions targeting this early-life window of programming.

## Data Availability

The data included in this article were extracted as published in the available original articles. No new data were generated to support this article.
